# Plasma biomarkers for predicting heart failure in patients with acute myocardial infarction

**DOI:** 10.5937/jomb0-50741

**Published:** 2025-01-24

**Authors:** Rong Guihong, Wang Xiao, Qin Xinling, Wang Yanxia, Quan Meilian, Xi Chunfeng

**Affiliations:** 1 Affiliated Hospital of Guilin Medical University, Department of Clinical Laboratory, Guilin, China

**Keywords:** heart failure, acute myocardial infarction, GDF-15, NT-proBNP, srčana insuficijencija, akutni infarkt miokarda, GDF-15, NT-proBNP

## Abstract

**Background:**

Heart failure (HF) following acute myocardial infarction (AMI) is characterized by high mortality and disability rates, which highlights the need of timely and effective revascularization. Recent studies suggested the potential predictivity of biomarkers NT-proBNP, cTNT, PLR, NLR, and GDF-15 in cardiovascular events, but their value in HF patients post-AMI still require further verification. Therefore, the present study aimed to identify potent biomarkers for prognosticating the development of HF subsequent to AMI, and to devise early interception and therapeutic strategies.

**Methods:**

A total of 170 patients with AMI were enrolled in this study, including 44 patients with post-AMI HF and 126 patients with post-AMI non-HF. We measured the serum biomarkers NT-proBNP, cTNT, PLR, NLR, and GDF-15 in all patients using enzyme-linked immunosorbent assay (ELISA). Subsequently, we performed t-tests to assess the predictive value of these biomarkers for post-AMI HF.

**Results:**

In the group of HF patients, cTNT, NT-proBNP, GDF-15 and NLR was significantly higher than in the group without HF, but PLR was not. The AUC of NLR to predict HF after AMI was 0.632 (95%CI: 0.542-0.723), P=0.010, with a cut-off value of (3.86×109)/L, GDF-15 was 0.661 (95%CI: 0.560-0.763), P=0.002, with a cutoff value of 1.35 ng/mL, and NT-proBNP was 0.82 (95%CI: 0.723-0.876), P<0.001, with a cut-off value of 1444 pg/mL.

**Conclusions:**

In five biomarkers, there is predictive value in NT-proBNP, NLR, GDF-15 for patients with HF after AMI.

## Abstract (sr)

**Uvod:**

Srčana insuficijencija (HF) nakon akutnog infarkta miokarda (AMI) karakteriše visoka stopa mortaliteta i invaliditeta, što ukazuje na potrebu pravovremene i efikasne revaskularizacije. Nedavne studije sugerišu potencijalnu prediktivnost biomarkera NT-proBNP cTNT, PLR, NLR i GDF-15 u kardiovaskularnim događajima, ali njihova vrednost kod pacijenata sa HF nakon AMI i dalje zahteva dalju verifikaciju. Stoga je ova studija imala za cilj da identifikuje močne biomarkere za prognozu razvoja HF nakon AMI, i da osmisli rano presretanje i terapijske strategije.

**Metode:**

Ukupno 170 pacijenata sa AMI je uključeno u ovu studiju, uključujuči 44 pacijenta sa HF nakon AMI i 126 pacijenata sa post-AMI ne-HF Merili smo serumske biomarkere NT-proBNP cTNT, PLR, NLR i GDF-15 kod svih pacijenata primenom enzimskog imunosorbentnog testa (ELISA). Nakon toga, uradili smo t-testove da procenimo prediktivnu vrednost ovih biomarkera za HF nakon AMI.

**Rezultati:**

U grupi pacijenata sa HF, cTNT, NT-proBNP, GDF-15 i NLR je bio značajno veći nego u grupi bez HF, ali PLR nije bio. AUC NLR za predviđanje HF nakon AMI je bio 0,632 (95%CI: 0,542-0,723), P=0,010, sa graničnom vrednošču (3,86x109)/L, GDF-15 je bio 0,661 (95%CI: 0,560-0,763), P=0,002, sa graničnom vrednošću od 1,35 ng/mL, a NT-proBNP je bio 0,82 (95%CI: 0,723-0,876), P<0,001, sa graničnom vrednošću od 1444 pg/mL.

**Zaključak:**

Kod pet biomarkera, postoji prediktivna vrednost NT-proBNP NLR, GDF-15 za pacijente sa HF posle AMI.

## Introduction

Heart failure (HF) is a common complication of acute myocardial infarction (AMI), with an incidenceranging from 14% to 36% and an in-hospital mortality rate of 30% to 50% [1] [2]. The conjunction of HF with AMI markedly escalates mortality risk, both during hospitalization and in the long term, where HF patients exhibit a two- to four-fold higher in-hospital mortality compared to those without HF. This heightened risk persists over short intervals, such as within 30 days, and extends into the long term [2]. The mortality in patients with AMI is significantly reduced and prognosis of those is improved recent years, but HF after AMI remains a problem which is affecting the survival of these patients [3]. The principal clinical approaches for managing HF subsequent to AMI include early initiation of angiotensin-convertingenzyme inhibitors and aldosterone antagonists, cautious implementation of beta-blockers to avoid early complications, mechanical circulatory supports, with the application of novel therapies such as angiotensin receptor-neprilysin inhibitor sacubitril/valsartan to further enhance outcomes [4] [5] [6] [7]. Although these approaches were shown to improve the prognosis of patients with HF, clinical treatments are still confronted with challenges in the early diagnosis of HF post-AMI. Prompt diagnosis and immediate treatment are crucial in HF post-AMI to mitigate disease progression and improve outcomes, as delays lead to increased risks [8]. Therefore, identifying new biomarkers or diagnostic tools holds the potential to enhance early detection, improve risk stratification, and facilitate tailored interventions, thereby mitigating adverse outcomes and advancing the precision medicine approach in this high-risk patient population.

Plasma biomarkers have the advantages of accessible in clinical and reflecting multiple pathologicmechanisms. Several plasma markers have been shown to predict HF after AMI [9]. There is a lack of objective biomarkers for early detection of HF in AMI patients. NT-proBNP, a traditional marker of HF, is commonly used to diagnose and predict prognosis in patients who are likely to develop HF. Study have reported that NT-proBNP is effective in predicting ventricular remodeling in patients with HF following AMI [10]. Interestingly, NT-proBNP is produced when cardiomyocytes are subjected to tension and pressure, and is mainly synthesized and secreted by ventricular myocytes [11]. But cardiomyocytes loss is an important cause of HF in patients after AMI [1], so the accuracy of NT-proBNP in predicting the degree of severity in early stages of HF post-AMI still require further verifications. GDF-15 is involved in myocardial injury and ventricular remodeling, and its serum level is an independent risk predictor of adverse cardiovascular events [12]. cTnT is a cardiac troponin that is able to detect minor heart muscle damage, and also applied in the diagnosis and prognosis of HF [13]. Likewise, PLR (lymphocyte to monocyte ratio) and NLR (neutrophil to lymphocyte ratio) are inflammatory markers that are expressed at high levels in a variety of cardiovascular diseases [14]. Study have suggested that GDF-15 independently and dynamically predicts risk of adverse outcomes at 1-year, and the multi-marker-model assessment of GDF-15, NT-proBNP, and troponin I also showed strong prediction value in acute HF [15]. But the effects of these markers in predicting HF in AMI patients still require further verification. Therefore, further investigating the prediction value of NT-proBNP, cTNT, PLR, NLR, and GDF-15 in HF post-AMI patients is needed.

In the present study, we aimed to search for effective biomarkers to predict the occurrence of HF after AMI, along with early prevention and treatment strategies. A total of 170 patients with AMI were enrolled and studied to investigate the levels of five markers: NT-proBNP, cTNT, PLR, NLR, and GDF-15 in predicting HF post-AMI.

## Materials and methods

### Study subjects

A total of 170 patients with AMI between June 2021 and June 2022 were enrolled in this study. The inclusion and exclusion criteria are based on previous reports [10].

Inlcusion criteria: (1) Participants must conform to the diagnostic guidelines for Acute ST-segmentElevation Myocardial Infarction, presenting with persistent chest pain exceeding 30 minutes, alongside abnormal elevations in myocardial injury markers such as creatine kinase or troponin levels, and exhibiting electrocardiogram (ECG) evidence of ST-segment elevation exceeding 0.1 mV in at least two contiguous leads. (2) Enrollees must meet the diagnostic criteria for post-infarction heart failure, classified under Killip classes II, III, or IV, adhering to the European Society of Cardiology (ESC) Guidelines for Acute ST-segment Elevation Myocardial Infarction management. (3) Candidates must secure approval from the institutional Ethics Committees, and written informed consent must be obtained from both the patient and their legal representatives.

Exclusion criteria: (1) Individuals with a documented history of myocardial infarction, pre-existing heart failure, or other cardiac disorders prior to the current event. (2) Patients presenting with significant comorbidities, including advanced diabetes, active malignancy, or infectious diseases that could confound study outcomes. (3) Those with impaired function of vital organs such as the liver, kidneys, thyroid, or any other systemic dysfunction that might influence the study results or management strategies. (4) Exclusion also applies to individuals diagnosed with connective tissue disease, autoimmune disease, or heart failure secondary to known conditions such as heart valve disease or cardiomyopathy with a clear cause. (5) Additional exclusion criterion encompasses patients whose heart failure diagnosis is based primarily on clinical manifestations alone, such as cardiac arrhythmias, pulmonary crackles, lower limb edema, distended jugular veins, hepatomegaly, or signs suggestive of pulmonary hypertension, without meeting the rigorous electrocardiographic and biochemical criteria specified for acute myocardial infarction.

### Laboratory measurements

Relevant clinical data: age, gender, history of hypertension, diabetes and smoke, left ventricular ejection fraction (LVEF) and left ventricular end-diastolic dimension (LVEDD).

Analysis of relevant indexes: 5 mL of fasted elbow venous blood was collected early in the morning for plasmaal indexes on the second day after the patient was admitted to the hospital, as follows: GDF-15 by enzyme-linked immunosorbent assay (ELISA), the kit was provided by Shanghai Yubo Biotechnology Co., Ltd. (Shanghai, China) and the operation procedures were strictly in accordance with the kit instructions. NT-proBNP and cTNT were detected by electrochemiluminescence immunoassay, using Roche automatic electrochemiluminescence analyzer and supporting reagents, and operated according to the standard operating procedures of the instrument. NLR and PLR: elbow venous blood was collected from patients on an empty stomach after admission, and the white blood cell count (WBC), platelet count (PLT), and lymphocyte count (Lym) in blood were detected using the Sysmex automatic hematology analyzer provided by the Laboratory Department of our hospital, and NLR and PLR were calculated. Other venous blood items included total cholesterol (TC), triglyceride (TG), creatine kinase-MB (CK-MB).

### Statistical analysis

The continuous variable is denoted as either the mean standard deviation or the median (interquartile range), while the categorical variable is presented as a count (percentage). Disparities among continuous variables were evaluated using independent t-tests for normally distributed data and Wilcoxon’s rank sum tests for non-normally distributed data. Chi-squared tests and Fisher’s exact tests were employed for the analysis of categorical variables. The diagnostic value of the indices was assessed using a receiver operating characteristic (ROC) web-tool. A P-value below 0.05 was deemed statistically significant.

## Results

### Clinical characteristics

A total of 170 people were included in this study, of whom 44 patients with HF after AMI, 31 men (70.5%), with a mean age of (67.4±14.3) years; 126 patients without HF, 99 men (78.0%), with a mean age of (62.4±15.4) years, no statistical difference was seen between the two groups in terms of gender, and the age of the HF group was higher than that of the group without HF, with statistical difference (P=0.050). In the HF group, there were more low hemoglobin in patients than in the HF group (P<0.001), and there was no statistical difference between the two groups for hypertension, diabetes mellitus, smoke, TC, triglyceride, Creatine kinase – MB, LVEF and LVEDD (See Table 1).

**Table 1:** Clinical characteristics of patients with HF after AMI and non-HF after AMI.

	HF (n=44)	Non-HF (n=126)	P value
Age (years)	67.4±14.3	62.4±15.4	0.050
Male (%)	31 (70.5)	99 (78.0)	0.315
Hypertension (%)	13 (29.5)	44 (34.9)	0.516
Diabetes mellitus (%)	9 (20.5)	18 (14.3)	0.335
Smoke (%)	4 (9.1)	7 (5.5)	0.404
TC (mmol/L)	4.07±1.17	4.39±1.12	0.119
TG (mmol/L)	1.60±1.19	1.51±0.91	0.621
HGB (g/L)	115.8±21.6	134.9±18.1	<0.001
CK-MB (U/L)	100.8±172.6	111.2±143.4	0.689
LVEF (%)	52.7±9.4	55.9±9.9	0.59
LVEDD (mm)	42.0±6.1	39.0±9.9	0.64

### Plasma biomarkers

In the group of HF patients, cTNT was significantly higher than in the group without HF, (2.87±2.90) ng/mL vs. (1.89±2.10) ng/mL, and the same results were found for NT-proBNP, 3961(1670-5239) pg/mL vs. 281 (50–1900) pg/mL and GDF-15, (1.28±0.47) ng/mL vs. (1.12±0.54)ng/mL. NLR was 6.93 (4.93–9.76) × 10^9^/L and 4.89 (2.73–8.90) ×10^9^/L, P=0.016, with significantly statistically difference. PLR was 198 (123–354) × 10^9^/L and 181 (133–273) × 10^9^/L, P=0.257, with no statistically difference (Table 2).

**Table 2:** Plasma biomarkers of patients with HF after AMI and non-HF after AMI.

	HF	non-HF	P value
Neutrophil (×10^9^/L)	7.86 (5.14–11.76)	6.28 (4.19–8.78)	0.083
Lymphocyte (×10^9^/L)	1.12 (0.81–1.50)	1.22 (0.81–1.75)	0.501
Platelet (×10^9^/L)	227 (182–333)	238 (186–291)	0.105
NLR	6.93 (4.93–9.76)	4.89 (2.73–8.90)	0.016
PLR	198 (123–354)	181 (133–273)	0.257
GDF-15 (ng/mL)	1.35±0.45	1.09±0.40	<0.001
cTNT (ng/mL)	2.87±2.90	1.89±2.10	0.018
NT-proBNP (pg/mL)	3961 (1670–5239)	281 (50–1900)	<0.001

### Predictive value of plasma biomarkers for HF after AMI

**Figure 1:**
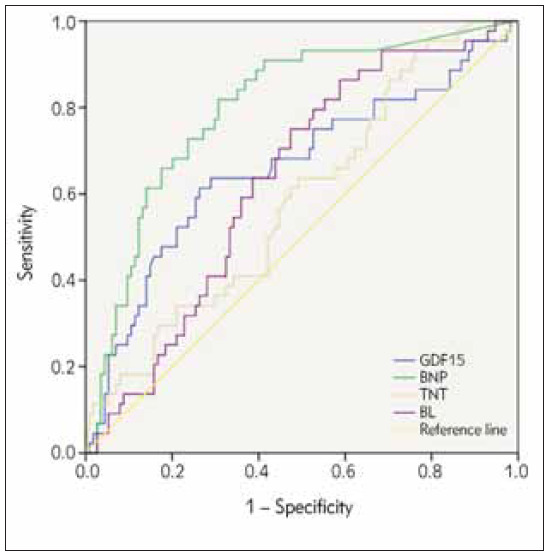
ROC curve of NLR, cTNT, GDF-15 and NTproBNP.

The AUC of NLR to predict HF after AMI was 0.632 (95%CI: 0.542–0.723), P=0.010, with a cut-off value of 3.86×109/L, GDF-15 was 0.661 (95%CI: 0.560–0.763), P=0.002, with a cut-off value of 1.35 ng/mL, and NT-proBNP was 0.82 (95%CI: 0.723–-0.876), P<0.001, with a cut-off value of 1444 pg/mL. While The AUC of cTNT is 0.587 (95%CI: 0.490–0.683), but no statistically difference (See Table 3 and Figure 1).

**Table 3:** Sensitivity, specificity and AUC of NLR, cTNT, GDF-15 and NT-proBNP.

	Se	Sp	AUC(95%CI)	P	Cut off
NLR	0.86	0.41	0.632 (0.542–0.723)	0.010	3.86
GDF-15 (ng/mL)	0.61	0.76	0.661 (0.560–0.763)	0.002	1.35
cTNT (ng/mL)	0.93	0.30	0.587 (0.490–0.683)	0.092	0.20
NT-proBNP (pg/mL)	0.82	0.72	0.800 (0.723–0.876)	<0.001	1444

## Discussion

AMI is the main cause of death in patients with cardiovascular diseases, and the developing of early reperfusion therapy, including thrombolysis and primary PCI, has significantly reduced mortality in these patients [16]. However, HF after AMI made poor prognosis in these patients, which cannot be ingored. with in-hospital death and mortality after 1 year significantly higher in patients who developed HF after infarction than in those who did not [17], and the same trend exists even in STEMI patients treated with primary PCI [18]. Therefore, accurate early identification of patients with HF after AMI is an important way to address this problem.

Post-infarction ventricular remodeling is an important cause of the development of HF, including early remodeling (2–3 weeks after infarction) and late remodeling (3–6 months after infarction) [9]. Gerber Y et al. [19] showed that more than half of the patients with infarction developed HF within 1 week of onset, and patients with early onset HF progressed more rapidly and had a worse prognosis. This study aims to identify HF patients early by analyzing the predictive value of serum marker levels, including NT-proBNP, cTNT, PLR, NLR, and GDF-15, in the patients with AMI.

The mechanisms include the following aspects myocardial cell loss, ventricular remodeling, neurohumoral system (SNS, RAAS) activation, and inflammatory injury. In this study, five plasma markers, NT-proBNP, cTNT, PLR, NLR, and GDF-15, were selected, on the one hand, serum markers have the advantage of being objective and easily available, on the other hand, they can be used to reflect different pathophysiological mechanisms from:

(1) BNP: NT-proBNP generation occurs in response to cardiac myocyte stretch and pressure, primarily by ventricular cells. Ischemia or injury-induced myocyte expansion leads to elevated NT-proBNP secretion, thereby raising plasma concentrations, indicative of heightened cardiac workload and dysfunction. Furthermore, NT-proBNP release is intricately tied to cardiac remodeling post-myocardial infarction. This multifaceted process encompasses myocardial hypertrophy, cell loss, and alterations in surviving cells, collectively deteriorating the left ventricular diastolic function and exacerbating or initiating heart failure [11]. Elevated BNP or NT-proBNPreflects increased ventricular wall stress and increased volume load and has been widely used to predict prognosis in patients with HF. In HF after AMI patients, NT-proBNP has also been shown to predict patient prognosis [20]. In the present study, NT-proBNP predicted HF in AMI patients. In our study, NT-proBNP is the marker with the highest predictive value, however, NT-proBNP can be elevated in a variety of conditions with low specificity.

(2) cTNT: When myocardial cells undergo damage due to ischemia (insufficient blood supply), cTnTis discharged from the impaired cardiac muscle cells. This release is typically associated with the demise of these cells, involving processes such as apoptosis, autophagy, and necrosis, among others. Even in the early stages of ischemia, where overt tissue necrosis may not be evident, recurrent and brief ischemic episodes can facilitate cTnT release. This is potentially linked to locally elevated calcium ion concentrations within the cells during ischemia. The rise in calcium levels activates calcium-dependent proteases like calpains, leading to chronic proteolytic degradation of myofibrillar proteins, including cTnT [21]. cTNT is used in AMI both as a diagnostic indicatorand as a predictor of prognosis [22]. Higher levels of cTNT reflect more cardiomyocyte loss, and segmental ventricular wall motion abnormalities leading to decreased cardiac output are the main mechanism for the development of HF in infarcted patients. But in this study cTNT cannot predicted HF patients with AMI.

(3) NLR and PLR: As new inflammatory markers, NLR and PLR can reflect systemic inflammatoryand immunosuppressive states. Higher NLR and PLR values were associated with more severe systemic inflammatory and immunosuppressive states. For example, in patients with acute decompensated heart failure with HFpEF, elevated NLR and PLR are associated with an increased risk of cardiac death. In addition, several studies have shown that NLR and PLR have independent value in predicting the prognosis of AMI patients, especially for assessing the risk of death in hospital [23] [24]. Previous studies have shown that NLR and PLR can predict the prognosis of patients with heart attack and HF [25], and NLR can also predict infarct size and adverse events in patients with heart attack. NLR and PLR as routine blood indicators have the advantages of being easily available and reproducible, and are more convenient to apply in clinical practice. In our study, NLR can be used to predicted HF patients with AMI, but PLR was no difference in two groups. The likely reason is that neutrophil cells recruitment due to inflammation is more pronounced than platelet aggregation in heart failure.

(4) GDF-15: GDF-15 plays a protective role in the heart, alleviating heart damage by inhibiting apoptosis, hypertrophy and adverse remodeling of cardiomyocytes. In addition, studies have shown that GDF-15 can promote the glycolysis and mitochondrial oxidation of cardiomyocytes, thereby increasing the production of ATP, so that the energy metabolism of cardiomyocytes tends to a compensatory state against the metabolic failure caused by the overactivation of the sympathetic nerve [26]. However, although GDF-15 has a certain protective effect, its high levels are also strongly associated with the development of heart failure. Previous studies have shown that GDF-15 predicts poor prognosis in HF patients and is independent of BNP [27]. Myrmel et al. [28] study confirmed that GDF-15 also has a prognostic role in acute heart attack patients. In this study we got the same result that GDF-15 could be used to predicted HF patients with AMI.

Other novel plasma markers have also been shown to predict the prognosis of patients with HF after AMI, such as macroendothelin-1 [29], lncRNA-NRF [30], and Sirtuin2 [31], but they are less commonly used in the clinic and need to be confirmed by further studies. plasma markers have the disadvantage of low specificity and susceptibility to multiple factors, and the combination of multiple indicators can improve the accuracy of prediction [32].

The study carries significant clinical implications, highlighting the importance of recognizing specific patient profiles at risk for HF following AMI. It underscores that older age and lower hemoglobin levels are associated with an increased likelihood of developing HF post-AMI, suggesting a need for heightened surveillance in these demographics. Furthermore, the research underscores the predictive potential of plasma biomarkers, particularly NT-proBNP, GDF-15, and NLR, in forecasting HF incidence. These findings can guide clinicians in early identification of high-risk AMI patients, enabling prompt intervention strategies to prevent or mitigate HF development. In sum, in addition to NT-proBNP, GDF-15, NLR can be used to predict the development of heart failure in patients with infarction. But this was not observed in cTNT and PLR in this study. NT-proBNP has high specificity and sensitivity as a traditional predictive marker of heart failure, but more markers that can be referenced are helpful in helping to risk stratify patients after hospital admission.

Looking ahead, future research avenues should encompass several key areas to further refine our understanding and management of HF post-AMI. Developing composite predictive models incorporating multiple biomarkers could enhance predictive accuracy and clinical utility. Investigating the temporal dynamics of these biomarkers and their correlation with HF progression would offer insights into disease trajectory. Additionally, delving into the underlying mechanisms connecting low hemoglobin to HF and exploring potential interventions targeting hemoglobin levels is warranted. Understanding sex- and agespecific differences in HF development, along with the relationship between biomarker profiles and treatment responses, opens avenues for personalized medicine. Lastly, elucidating the effectiveness of tailored therapeutic interventions guided by these biomarkers will be pivotal in improving outcomes and reducing HF-related morbidity in AMI survivors.

## Conclusions

The present study contributes valuable clinical insights into the demographic characteristics and biomarker profiles associated with heart failure development following acute myocardial infarction. In five biomarkers, there is predictive value in NT-proBNP, GDF-15, NLR for patients with HF after AMI but not cTNT and PLR. The AUC of NLR to predict HF after AMI was 0.632 (95%CI: 0.542–0.723), with a cut-off value of (3.86×10^9^)/L, GDF-15 was 0.661 (95%CI: 0.560–0.763), with a cut-off value of 1.35 ng/mL, and NT-proBNP was 0.82 (95%CI: 0.723–0.876), with a cut-off value of 1444 pg/mL. By identifying older age, low hemoglobin levels, and elevated concentrations of NT-proBNP, GDF-15, and NLR as significant predictors, it paves the way for enhanced risk stratification and targeted surveillance in post-AMI patients. However, the study’s limitations necessitate cautious interpretation of the results and emphasize the imperative for further investigation. Firstly, the sample size of 170 participants, though informative, may not fully represent the broader population, limiting the generalizability of findings. The statistical difference in age between the HF and non-HF groups, despite being significant, necessitates cautious interpretation due to potential confounding effects of age on outcomes. Secondly, while biomarkers like NT-proBNP, GDF-15, and NLR demonstrated predictive abilities, the lack of statistical significance for cTNT— a commonly used marker for myocardial damage—underscores the complexity of HF pathophysiology and suggests the involvement of multifactorial mechanisms yet to be fully elucidated. This discrepancy raises questions about the optimal panel of biomarkers for HF prediction and calls for refined biomarker selection or the exploration of novel markers. Lastly, the study did not extensively explore the impact of comorbidities such as hypertension, diabetes mellitus, and smoking on the development of HF post-AMI, even though these factors are known to influence cardiovascular health. Future research should incorporate comprehensive assessments of these comorbidities and their interactions with the identified biomarkers.

## Dodatak

### Ethical compliance

This study was approved by the ethics committee of Affiliated Hospital of Guilin Medical University. Signed written informed consents were obtained from the patients and/or guardians.

### Author contributions

GR, XW and CX designed the study and performed the experiments, XQ and YW collected the data, XQ, YW and MQ analyzed the data, GR, XW and CX prepared the manuscript. All authors read and approved the final manuscript.

### Funding

This work was supported by the Research Project funded by Health Commission of Guangxi Zhuang Autonomous Region (No. Z20210121).

### Conflict of interest statement

All the authors declare that they have no conflict of interest in this work.

### Contribution

Guihong Rong and Xiao Wang contributed equally to this work.
